# Effect of pH on the structure and drug release profiles of layer-by-layer assembled films containing polyelectrolyte, micelles, and graphene oxide

**DOI:** 10.1038/srep24158

**Published:** 2016-04-07

**Authors:** Uiyoung Han, Younghye Seo, Jinkee Hong

**Affiliations:** 1 School of Chemical Engineering & Materials Science, College of Engineering, Chung-Ang University, 47 Heukseok-ro, Dongjak-gu, Seoul 156-756, Republic of Korea

## Abstract

Layer by layer (lbl) assembled multilayer thin films are used in drug delivery systems with attractive advantages such as unlimited selection of building blocks and free modification of the film structure. In this paper, we report the fundamental properties of lbl films constructed from different substances such as PS-*b*-PAA amphiphilic block copolymer micelles (BCM) as nano-sized drug vehicles, 2D-shaped graphene oxide (GO), and branched polyethylenimine (bPEI). These films were fabricated by successive lbl assembly as a result of electrostatic interactions between the carboxyl group of BCM and amine group of functionalized GO or bPEI under various pH conditions. We also compared the thickness, roughness, morphology and degree of adsorption of the (bPEI/BCM) films to those in the (GO/BCM) films. The results showed significant difference because of the distinct pH dependence of each material. In addition, drug release rates of the GO/BCM film were more rapid those of the (bPEI/BCM) film in pH 7.4 and pH 2 PBS buffer solutions. In (bPEI/BCM/GO/BCM) film, the inserted GO layers into bPEI/BCM multilayer induced rapid drug release. We believe that these materials & pH dependent film properties allow developments in the control of coating techniques for biological and biomedical applications.

The fabrication of multilayer thin films using layer-by-layer (lbl) assembly has been studied over the past several years. The multilayer thin films are formed by the successive adsorption of diverse materials on the surface of a film through various methods such as dipping, spraying, and spin coating^1^,^2^,^3^. The primary driving force in lbl assembly is the electrostatic interactions between oppositely charged materials^4^. In addition, the interfaces between the films also interact via hydrogen bonding^5^, covalent bonding^6^, and biospecific interaction^7^ among various functional groups. The lbl assembled films have great advantages in thin film technology. For instance, unlimited and versatile materials like polyelectrolytes, micelles, graphene oxide (GO), nanoparticles, proteins, and polysaccharides can be used as building blocks for fabricating lbl assembled films^8^,^9^,^10^,^11^,^12^. Also, structure and properties of lbl films can be easily controlled by altering the assembly conditions such as composition of solvents, pH, and salt concentration^13^,^14^,^15^. Lastly, lbl assembly technic is inexpensive and uncomplicated coating method. Therefore, lbl assembled films have been used in various application including electronic devices^16^, drug delivery carriers and biosensor^17^,^18^. In particular, films loaded with biomaterials such as antitumor agents, DNA, RNA, enzymes, and growth factors have been utilized as means to coat in biomedical devices^19^,^20^,^21^,^22^,^23^.

Especially, a multilayer thin film has a lot of potential as drug delivery carrier because it can be utilized for stimuli-responsive carrier^24^ and specific targeted delivery^25^. Among the various lbl assembly building blocks, polyelectrolytes have been most widely used because of facile method for controlling polymer properties such as surface charge density and structure. Moreover, drug release from polymer films can be controlled by controlling the porosity, dissociation, degradation, and crosslinking of the polymer layers^26^,^27^. However, polymer films used for drug delivery have some limitations such as the relatively low quantity of drugs that can be loaded on the films and decrease activity of the drugs by exposure to specific solvents. Therefore, BCM loaded with drugs in the core and corona have generally been utilized in drug delivery carriers^28^,^29^. Also, Xi Zhang and co-workers were beautifully incorporated block copolymer micelle (BCM) as a building block of lbl film for drug container^11^. However, multilayer films consisting of BCM are too brittle and rough to form freestanding films for drug delivery applications.

On the other hand, it is well known that carbon based materials have high electric, thermal and mechanical performances. Zhao, Xin, *et al*. fabricated PVA/GO film by lbl assembly and it had well-dispersed surface and high degree of planar orientation^10^. Commonly, graphene oxide-based multilayer films has improved Young’s modulus and tensile strength^30^. So, the graphene oxide layer enhances film hardness and flexibility and these application is mostly electric device. However, many research have been studied in past years about using graphene oxide sheets for good mechanical properties of drug delivery carriers and controlling the drug release rate. Liu *et al*. fabricated PEGylated graphene oxide based anticancer drug carrier without evident cytotoxicity^31^. Hong *et al*. demonstrated that ovalbumin release from in multilayer thin films could be controlled by inserted GO sheets^32^.

Consequently, multilayer films fabricated with these materials such as polymer/GO^10^, polymer/BCM^11^ and GO/BCM^33^ have been studied. However, there are few fundamental approaches (e.g., film growth, surface morphology, adsorption, release rate etc.) for fabricating thin films consisting of different structured materials such as 2-dimensional (2D)-GO sheets, 0D-micelle, and random-coil shaped polymers. And the pH condition is one of many factors that control these film properties.

In order to study the pH dependence of the thickness and morphology of the lbl assembled multilayer films, we synthesized coumarin 6 (C6, model drug) loaded polystyrene*-b-*poly(acrylic acid) (PS*-b-*PAA) BCM with positively charged GO. Subsequently, we fabricated bPEI/BCM and GO/BCM multilayer films under various pH conditions (Fig. 1). Following this, we investigated the difference in the quantity of BCM adsorbed on the bPEI and GO layers using a quartz crystal microbalance (QCM). Finally, we analyzed the effect of different structures between bPEI and GO on the release rate of the model drug from our films.

It is well known that the pH values influence to degree of ionization of weak polyelectrolytes^15^. Also, it has an impact on the assembly aspect of lbl multilayer films since it make a difference of the surface charge density of weak polyelectrolytes. Therefore, the properties of the lbl assembled films composed weak polyelectrolytes is significant in the pH of the dipping solution. Generally, when the pH value of the dipping solution is near to its pKa value, the film thickness is increased. Likewise, assembly nature of our films was similar to reported investigation. However, the change of the degree of ionization of weak polyelectrolytes by the pH condition differently effected to our film properties since bPEI, BCM, and GO had their specific chemical properties.

The controlling of film properties is important to modeling of drug delivery film. For instance, the film with high porosity and roughness had large surface area, thereby improving permeation of solvent^34^. Also, the decrease of deposited micelle on the film and the uniformed film surface enhanced the mechanical performance of the film like freestanding film^35^. For these reason, we believe that this study of pH dependent formation and dissociation of multilayer films using polyelectrolyte, BCM and GO as building blocks would provide the controlling technique of the properties of lbl assembled film which adapted to various purpose of biomedical applications.

## Methods

### Materials

Amphiphilic block copolymer, polystyrene_42K_*-b-*poly(acrylic acid)_4.5K_ (PS*-b-*PAA) was supplied by Polymer Source Inc. Branched polyethylenimine (bPEI, Mw = 25K), graphite powder (<20 μm), ethylenediamine (Mw = 60.1), C6 (Mw = 350.44), N-(3-dimethylaminopropyl)-N′-ethylcarbodiimide hydrochloride (EDC, Mw = 191.70), and all the other solvents were purchased from Sigma-Aldrich.

### Synthesis of C6 loaded BCM

Details on the formation of BCM with PS*-b-*PAA have already been investigated^9^. In addition, methods for the preparation of amphiphilic BCM loaded with hydrophobic drugs in the core have been reported^36^. Simply, a solution of PS_42K_*-b-*PAA_4.5K_ and C6 was prepared in N,N-dimethylmethanamide (DMF) at concentrations of 25 mg/mL and 2 mg/mL. Subsequently, 2 mL of the solution dissolved in C6 and BCM was added to 48 mL of water (pH 10) with vigorous stirring. As a result, the micelle consisted of a hydrophobic core with PS and C6 and a hydrophilic corona shell with PAA. After 6 h of additional stirring, the resulting solution was subjected to 48 h of dialysis in water to remove the remaining DMF.

### Synthesis of GO and ethylenediamine functionalized GO

We synthesized GO from graphite powder using a modified Hummers method^37^. The GO surface was then modified using ethylenediamine in order to obtain positively charged GO^38^. The ethylenediamine functionalized GO (referred to as positively charged GO(+), hereafter) solution had a pH of 8.4. The thickness and size of graphene oxide from graphite by modified hummer’s method are approximately 0.7 ~ 1.2 nm and 10 nm ~ 1 μm^39^. Also, the difference of size and thickness between GO and ethylenediamine functionalized GO are not noticeable^38^.

### Preparation of substrates and solutions

We fabricated the multilayer thin films on a silicon wafer by the lbl assembly method using GO, bPEI, and BCM solutions. First, the surfaces of the silicon wafer substrates were negatively charged by a 2 min oxygen plasma treatment. The concentrations of the bPEI, GO, and BCM solutions for lbl assembly were set at 1.0 mg/mL. The pH values of the bPEI and GO dipping solutions were varied from 7.0 to 9.0 and that of BCM was varied from 5.5 to 8. These pH conditions influenced the ionization of amine and carboxyl groups and the surface charge density of the materials used^40^,^41^. The pH values of the solutions were controlled using 0.1 M HCl and 0.1 M NaOH solutions.

### Fabrication of multilayer thin films by lbl assembly

The treated silicon substrates were first dipped into the bPEI solution for 10 min, rinsed three times in water (maintained at the same pH as the bPEI solution), and dried in air. The substrates were then dipped into the BCM solution for 10 min, following which they were rinsed and dried again in the same manner. This single cycle produced a single bilayer of bPEI and BCM, expressed as (bPEI/BCM)_1_. The dipping cycle was repeated until the desired number of bilayers was achieved. The GO/BCM and bPEI/BCM/GO/BCM multilayers were also obtained in a similar fashion. The resulting multilayer thin films were denoted as (bPEI/BCM)_n_, (GO/BCM)_n_, and (bPEI/BCM/GO/BCM)_m_, where n and m denote the number of bilayers and quadruple-layers. In (bPEI/BCM/GO/BCM)_m_ film, adsorption of each layer was identified by contact angle (Figure S3). All of the films were fabricated at 25 °C.

### Characterization of the multilayer thin film surface

Film thicknesses and roughness of the multilayer thin films fabricated under various pH conditions were measured with surface profilometry (Dektak 150, Veeco). In order to compare the effect of pH on the surface morphology and roughness of the films, the films were examined by atomic force microscopy (AFM, NX-10, Park Systems) and field-emission scanning electron microscopy (FE-SEM, LIBRA 120 microscope, Carl Zeiss). The AFM images were analyzed using Gwyddion and XEI software. The SEM images were obtained at an acceleration voltage of 5 kV.

### QCM analysis

The amount of each material adsorbed onto the film surface during lbl assembly was analyzed using QCM (QCM200, Stanford Research Systems). The multilayer thin films were prepared on a Au-chrome electrode using the lbl assembly method described earlier and the change in frequency of the quartz crystal was monitored continuously during the adsorption process. The masses of bPEI, GO, and BCM adsorbed were determined from the change in frequency of the QCM crystal using the Sauerbrey equation^42^.


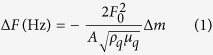


In the above equation, F_0_ (approximately 5 MHz) is the fundamental resonance frequency of the crystal, A is the area of the Au-chrome electrode, and μ_q_ (2.95 × 10^11 ^g/(cm·s^2^)) and ρ_q_ (2.65 g·cm^−3^) are the shear modulus and density of quartz, respectively. Applying these numerical values, the equation can be simplified as follows:


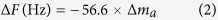


where ∆m_a_ is the mass change per unit area of the quartz crystal in μg/cm^2^.

### Drug release profile of the multilayer thin films

For determining the release profile of C6 (model drug), the multilayer thin films (ca. 0.4 × 0.4 cm) were immersed in 10 mL of phosphate buffered saline (PBS, 1X) buffer added to ethanol (EtOH; PBS/EtOH = 2:1) at a certain pH, for a predetermined length of time. Because the hydrophobic C6 was insoluble in PBS, ethanol added in PBS buffer. To prevent evaporation of ethanol, the release sample was stored in 1.5 mL Eppendorf tube at 4 °C. Subsequently, 0.1 mL of the sample was withdrawn at predetermined intervals of time. The fluorescence spectra of the C6 released into the PBS buffer were measured by a microplate reader (Synergy H1 Hybrid Multi-mode Microplate Reader, Bio-tek, USA). The concentrations of C6 were determined from calibration curves calculated from the fluorescence emission (λ_ex_ = 443 nm, λ_max_ = 520). The PBS buffer was placed in an incubator at 37 °C during the release process. In addition, we measured the decrease of the thickness of each film in DI water, pH 7.4 PBS/EtOH, and pH 2 PBS/EtOH during 72 hour incubating at 37 °C to investigate the film stability and the dissociation of multilayers.

## Results and Discussion

### Characterization of the films and solutions

The corona of the PS*-b-*PAA BCM bears a negative charge owing to the carboxyl group in PAA (COO^−^), whereas the functional groups of bPEI and GO bear positive charges owing to the amine group (NH_3_^+^). Multilayer thin films were assembled by the electrostatic interactions between the carboxyl group and amine group during repeated dipping in the solutions. To investigate the pH dependence of the lbl assembled multilayer films, two solutions with different pH values (from 5.5 to 9) were prepared. The first solution was in the fully ionized condition, whereas the other solution was half-ionized. Typically, the surface charge density of polyelectrolytes is induced by the ionization of functional groups such as carboxyl and amine groups^43^. Since the carboxyl groups of PAA under pH 5.5 and pH 8 conditions ionize partially (50%) and mostly (pK_a_ ≈ 5.5–6.5), respectively^44^, the pH of the C6 loaded PS*-b-*PAA micelle solutions were set at these values. In addition, the amine groups in GO and bPEI at pH 9 and pH 7 ionize almost 50% and mostly (pK_a_ ≈ 8.2–9.0), respectively^40^,^45^. Therefore, the GO and bPEI solutions were maintained at pH 9 and pH 7 respectively.

### Thickness of bPEI/BCM multilayer films

**Figure 2:**
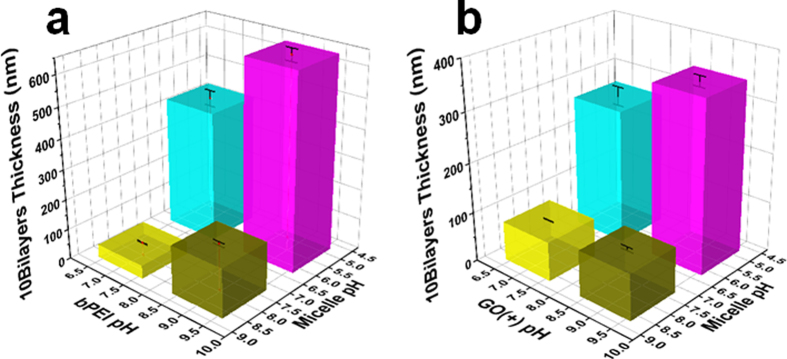
Thicknesses of (**a**) (bPEI/BCM)_10_ and (**b**) (GO/BCM)_10_ films fabricated in bPEI, GO, and BCM solutions under different pH conditions.

The number of bilayers for all the films was set at 10. However, the bPEI/BCM and GO/BCM multilayer films assembled under different pH conditions showed different thickness trends (Fig. 2), since the thickness is affected by the pH of the dipping solution. The thicknesses of the (bPEI/BCM)_10_ films prepared from pH 5.5 BCM/pH 9 bPEI and pH 5.5 BCM/pH 7 bPEI solutions were 640.7 ± 20.7 and 429.7 ± 25.9 nm, respectively. Meanwhile, the thicknesses of the (bPEI/BCM)_10_ films prepared from pH 8 BCM/pH 9 bPEI and pH 8 BCM/pH 7 bPEI solutions were 159.3 ± 6.2 and 43.7 ± 2.1 nm, respectively (Fig. 2a). In other words, when the pH of the bPEI solution was changed from 9 to 7, the film thickness decreased from 640.7 nm to 429.7 nm (32.9%) with the pH 5.5 BCM solution and from 159.3 nm to 43.7 nm (72.5%) with the pH 8 BCM solution. However, when the pH of the BCM solution was changed from 5.5 to 8, the film thickness decreased from 640.7 nm to 159.3 nm (75.0%) with the pH 9 bPEI solution and from 429.7 nm to 43.7 nm (89.8%) with the pH 7 bPEI solution.

Normally, electrostatic attraction and repulsion interactions are increased by high surface charges. However, all of the film thickness at fully- ionized pH conditions is lower than at half-ionized pH conditions. It means that repulsion more affects the film thickness than electrostatic interaction when it is compared at fully ionized and half-ionized pH conditions. Also, based on these film thickness variations, we can conclude that the change in the pH of the BCM solution more significantly impacts the film thickness than the change in the pH of the bPEI solution, because the diameter of BCM is nearly 52 nm (Figure S1) larger than the thickness of the bPEI layer. And the repulsion of BCM impacts the film thickness more than the structural transition of the polymer.

On the other hand, the pH of the dipping solution affects not only the ionization of the materials in solution but the ionization of the deposited materials. It is important fact to fabricate lbl assembled multilayer because deposited layer can be swollen and denatured. The degree of the ionization of carboxyl group in the PS-b-PAA micelle at pH 2 and that of amine group in bPEI at pH 11 are zero, thereby occurring morphological denaturation of deposited layer in bPEI/BCM film at these pH conditions. But the deposited layer of our films are hardly affected because all of the pH conditions are 5.5, 7, 8, and 9. In this case, the surface charge density of deposited bPEI layer at immersing in the pH 5.5 BCM solution is higher than in the pH 8 BCM solution. So, the pH 5.5 BCM is more adsorbed on bPEI layer than the pH 8 BCM because the interaction is increased by surface charge density of deposited layer. (as mentioned above, the pH 8 BCM has high repulsion) However, the degree of ionization of deposited BCM layer is hardly changed since the carboxyl group in BCM fully ionized at the pH 7 and 9 bPEI solutions.

In addition, the film thickness is more significantly influenced by the pH of the bPEI solution when the BCM solution is at pH 8, than when it is at pH 5.5 (32.9% < 72.5%), because the proportion of the bPEI layer in the overall film is higher at pH 8 than at pH 5.5. Likewise, the influence of the pH of the BCM solution on the film thickness is greater when the bPEI solution is at pH 7 than when it is at pH 9 (89.8% > 75.0%), since the proportion of the BCM layer in the overall film is higher when the BCM solution is at pH 8 than when it is at pH 5.5.

### Thickness of GO/BCM multilayer films

In the case of the GO/BCM multilayer films (Fig. 2b), the film thicknesses were calculated as 345.4 ± 10.7 nm (pH 5.5 BCM/pH 9 GO), 269.6 ± 18.9 nm (pH 5.5 BCM/pH 7 GO), 92.0 ± 3.8 nm (pH 8 BCM/pH 9 GO), and 78.7 ± 1.0 nm (pH 8 BCM/pH 7 GO) for the same pH combinations of bPEI/BCM films. In other words, we find the same remarkable effect of pH of the BCM solution on the thickness trend of the GO/BCM films. On the contrary, the difference in the film thickness under same pH of BCM solution (each 5.5 and 8) was less influenced by the pH change of the GO solution (92.0 nm → 78.7 nm (14.4%), 345.4 nm → 269.6 nm (21.9%)) than that of the bPEI solution (159.3 nm → 43.7 nm (72.5%), 640.7 → 429.7 nm (32.9%)). This discrepancy is attributed to the difference in structures between GO and bPEI. If the surface charge density of weak polyelectrolytes such as bPEI is decreased or increased by salt screening, ionization, and protonation, the polyelectrolyte chains transition into a coil-shape with decreasing charge density or to a linear-shape with increasing charge density^46^. In contrast, the phenomenon of surface expansion is not observed in 2D GO sheets^47^. On the other hand, variation in the pH of the BCM solution is effective in changing the GO/BCM film thickness, as in the case of the bPEI/BCM films.

Also, the change of the surface charge density of the deposited GO layer is similar to bPEI/BCM case. In both case, the adsorbed BCMs on film is influenced by repulsion with other BCM and interaction with deposited layer.

### AFM images

**Figure 3:**
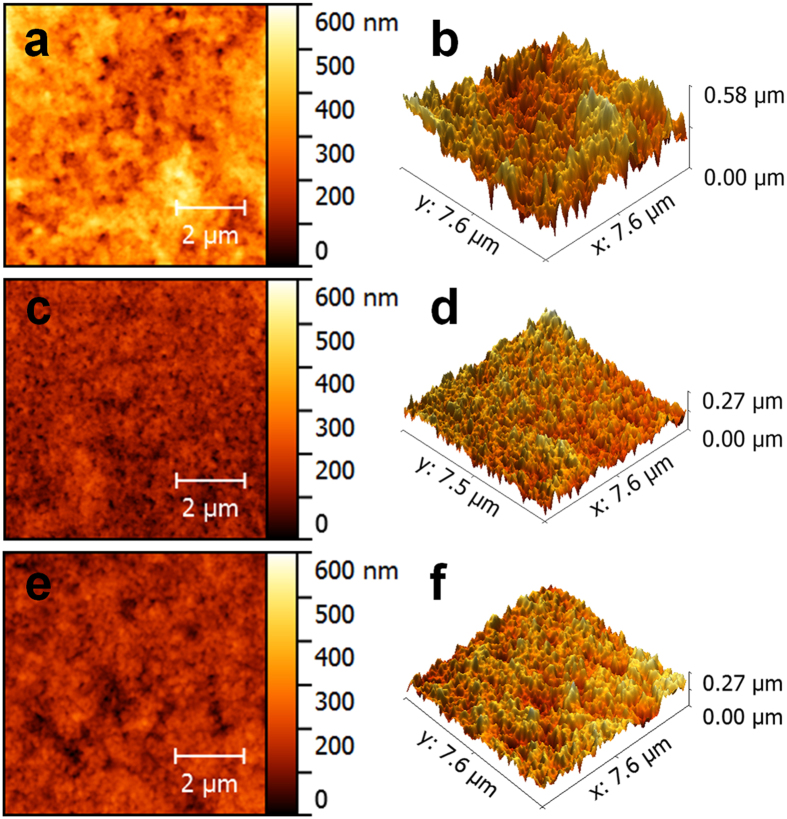
AFM images of the (bPEI/BCM)_7_ films (**a**,**b**) and (GO/BCM)_7_ films (**c**,**d**) fabricated in pH 5.5 BCM and pH 9 GO and bPEI solutions. (**e**,**f**) AFM image of a (GO/BCM)_18_ film fabricated in pH 8 BCM and pH 9 GO solutions.

AFM images of the (bPEI/BCM)_7_, (GO/BCM)_7_, and (GO/BCM)_18_ multilayer thin films showed the surface roughness of the films (Fig. 3). First, the surface roughness was expressed by the value of R_q_ (RMS, root mean square roughness), which was calculated by equation (3) 48. The parameter n in the equation corresponds to the number of intersections of the profile at the mean line and y represents height variations.


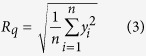


The calculated R_q_ values of the (bPEI/BCM)_7_ and (GO/BCM)_7_ multilayer films fabricated from the pH 9 bPEI, pH 9 GO, and pH 5.5 BCM solutions, are 76 and 33 nm (Fig. 3a–d), respectively. This implies that the layers of BCM adsorbed on GO are more uniform than those adsorbed on bPEI. Generally, 2D-structured GO sheets have flatter surfaces than chain-structured polymer layers. Moreover, the adsorption of 0D-structured micelles on GO and polymer layers intensifies this gap. In addition, we fabricated GO/BCM)_18_ multilayer films from pH 9 GO and pH 8 BCM solutions. In order to investigate the effect of BCM adsorption on the surface roughness of the GO/BCM multilayer film, we compared this film with the (GO/BCM)_7_ film fabricated from pH 9 GO and pH 5.5 BCM solutions. The thicknesses of both the films were approximately 280 nm, whereas the R_q_ values of the films were almost identical at 33 and 35 nm, respectively (Fig. 3c–f). Therefore, the surface roughness of the GO/BCM film was more significantly influenced by the entire thickness of the film than the change in BCM adsorption alone

### SEM images

**Figure 4:**
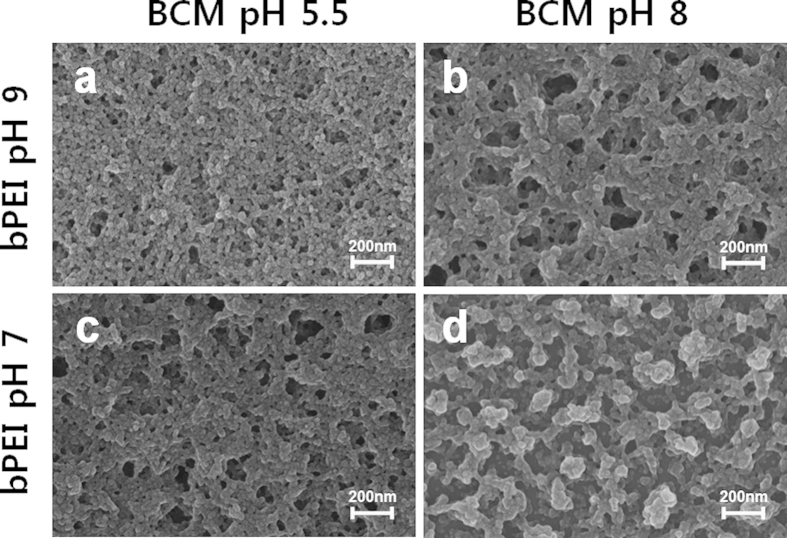
SEM images of the (bPEI/BCM)_10_ films fabricated under different pH conditions.

**Figure 5:**
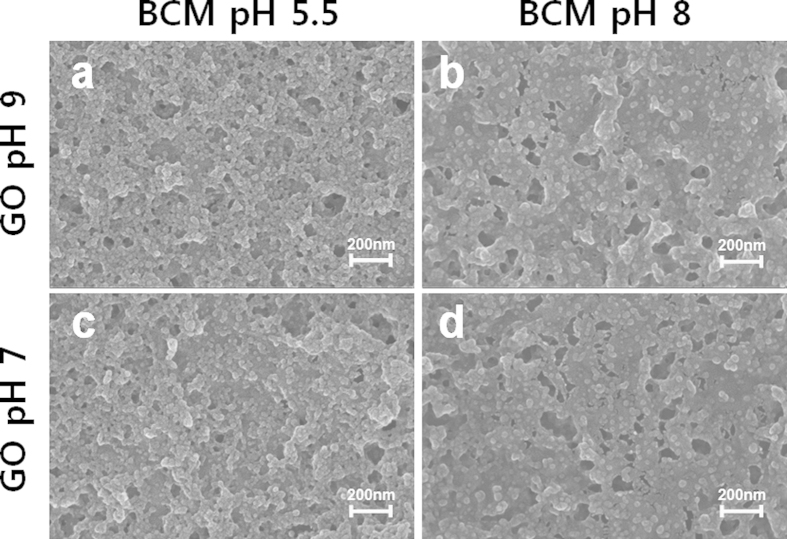
SEM images of the (GO/BCM)_10_ films fabricated under different pH conditions.

The SEM images shown in Fig. 4 reveal the morphologies of the bPEI/BCM and GO/BCM multilayer thin films, respectively. Randomly adsorbed BCMs on the bPEI or GO layers were observed on all the film surfaces. SEM image (Fig. 4a) of the film fabricated using pH 9 bPEI and pH 5.5 BCM solutions showed highly packed BCM. In contrast, when the pH of the bPEI and BCM solutions were changed from 9 to 7 and 5.5 to 8, respectively, the SEM images (Fig. 4b–d) revealed porous film surfaces. In particular, the highest surface porosity was obtained in the film fabricated from pH 8 BCM and pH 7 bPEI solutions. Owing to the high surface charge density of bPEI or BCM as a result of complete ionization, the polymer chain was transformed to the linear shape and the micelle particles repulsed each other. Consequently, the adsorption of the bPEI and BCM films was interrupted and the number of pores on the film surface was increased. However, the porosity of the GO/BCM films was slightly different when the pH of the GO solution was changed from 9 to 7 (Fig. 5a,c). Since the surface charge density of the GO sheet has less of an impact on the structure of the GO sheet as described earlier, the morphology of the GO/BCM film was influenced to a lesser extent by the pH change of the GO solution. In contrast, we observed significant difference in the amount of BCM adsorbed on the GO sheet from the pH 8 BCM solution (Fig. 5b,d). At pH 8, the adsorption of BCM is obstructed to a greater extent by the repulsion of highly charged BCM. In particular, this affects the GO/bPEI multilayer films more significantly than the bPEI/BCM films, because the BCM adsorbs more onto wide surfaces and the numerous amine groups of bPEI than GO.

### QCM analysis

**Figure 6:**
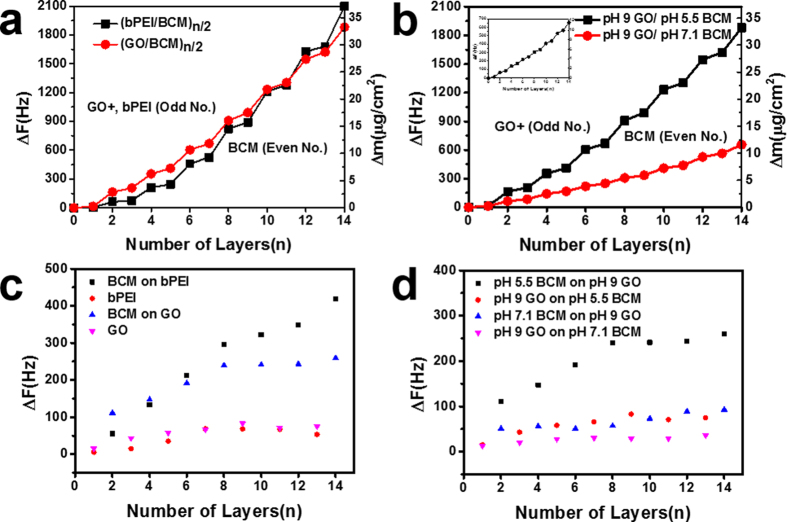
QCM graphs showing frequency change as a function of the number of layers in (**a**) (bPEI/BCM)_7_ and (GO/BCM)_7_ films (**b**) (GO/BCM)_7_ film fabricated at different pH values of BCM. The inset in (**b**) shows the QCM graph for the (GO/BCM)_7_ film fabricated in pH 7.1 BCM at a low scale, to compare with the variation in frequency change of the (GO/BCM)_7_ film fabricated in pH 5.5 BCM. (**c**,**d**) graphs show the frequency variation of bPEI, GO, and BCM in (**a**,**b**) graphs.

We analyzed the adsorption behavior of bPEI, GO, and BCM in the bPEI/BCM and GO/BCM multilayer films using QCM. Figure 6a shows the increase in the amount of bPEI/BCM and GO/BCM deposited as a function of the number of layers. The total amounts of the films adsorbed were approximately 33.2 and 37.1 μg/cm^2^, respectively, implying that the difference in the total masses of the bPEI/BCM and GO/BCM films was insignificant. Meanwhile, the alternating adsorption of bPEI, GO, and BCM layers resulted in an exponential increase in the frequency change, 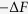, demonstrating that the amount of materials adsorbed per layer increased rapidly. In particular, the frequency changes of BCM (even number of layers) was higher than those of bPEI or GO (odd number of layers) and the growth curve of the bPEI/BCM multilayer increased more dramatically than that of the GO/BCM multilayer film. This resulted from two reasons: (1) the dominant material in the entire film was BCM and (2) the bPEI/BCM multilayer exhibited high coverage per layer. Additionally, as the number of layers increased from 2 to 14, the frequency change in the case of BCM adsorbed on bPEI increased relatively rapidly from 55.6 Hz to 418.6 Hz, whereas in the case of BCM adsorbed on GO, the frequency change increased from 111.5 Hz to 259.3 Hz (Fig. 6c). This difference caused relatively rapid growth of the bPEI/BCM film during lbl assembly. Next, we investigated the effect of pH change of BCM on the adsorption quantity of BCM on the GO layer. In Fig. 6b, the total adsorbed amount of the GO/BCM film at pH 8 of the BCM solution (11.6 μg/cm^2^) was less than half of that of the GO/BCM film for pH 5.5 BCM (33.2 μg/cm^2^). On the other hand, as shown in the inset of Fig. 5b, the growth curves of the GO/BCM films were linear and significant frequency changes occurred during the adsorption of BCM. However, when the number of adsorption layers increased from 2 to 14, the frequency change of pH 5.5 BCM adsorbed on the GO layer increased relatively rapidly from 111.5 Hz to 259.3 Hz and that of pH 7.1 BCM increased from 51.2 Hz to 92.7 Hz (Fig. 6d). This difference was due to the strong influence of the pH change of BCM on the adsorption quantities of BCM.

### Release profile

**Figure 7:**
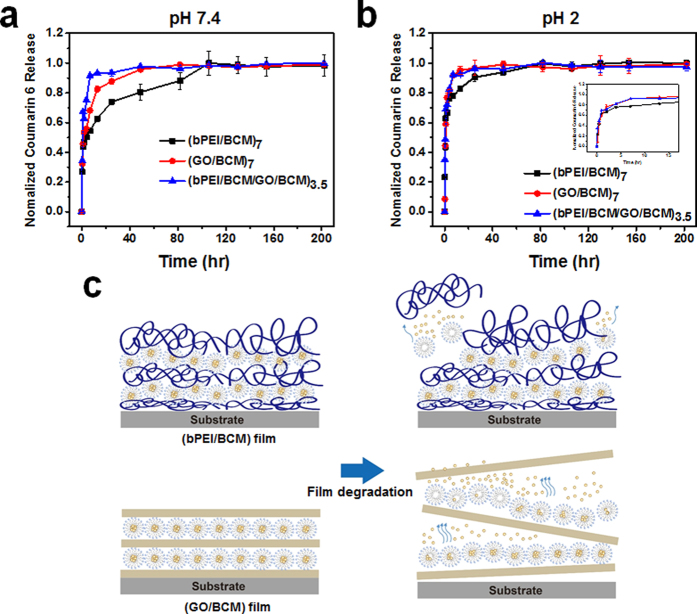
Release profile of coumarin 6 from (bPEI/BCM)_7_, (GO/BCM)_7_, and (bPEI/BCM/GO/BCM)_3.5_ films in PBS buffer containing ethanol (2:1 PBS/EtOH) at (**a**) pH 7.4 and (**b**) pH 2. All the release profiles were measured at 37 °C in an incubator and normalized to the fluorescence intensity measured under controlled conditions. Illustration (**c**) shows that BCM adsorbed on GO sheet more rapidly release C6 than on bPEI layer because of different degradation tendency between bPEI and GO.

The release of C6 (model drug) from the (bPEI/BCM)_7_, (GO/BCM)_7_, and (bPEI/BCM/GO/BCM)_3.5_ films (ca. 0.4 × 0.4 cm) was evaluated by immersing these films in PBS buffer under different pH conditions. The amount of C6 released was determined by fluorescence emission. The amounts of C6 released for the (bPEI/BCM)_7_, (bPEI/BCM/GO/BCM)_3.5_, and (GO/BCM)_7_ films were found to be 151.1 ± 2.6, 84.8 ± 1.6, and 47.8 ± 2.1 ng/mL, respectively (Figure S4). Typically, the degree of ionization of the carboxyl groups is influenced by pH variation, which causes variation in the surface charge density, as described in section 3.1. Therefore, we expected our films to be more easily eroded under acidic conditions (pH 2) than under weak basic conditions (pH 7.4), because the dominant driving force for lbl assembly in our multilayer films was electrostatic interactions, which was diminished by the deionization of carboxyl groups. Also, because of salt in PBS buffer, the structure of the BCM was denatured and the interaction between bPEI, GO and BCM layer was decreased. Actually, the thickness of these film decreased by 2 ~ 3%, 18 ~ 27% and 62 ~ 73% in DI water, pH 7.4 PBS/EtOH and pH 2.0 PBS/EtOH from the initial state (Figure S5). While the film remained partial after 72 hour, the C6 was almost released out at that period in the pH 7.4 and pH 2 conditions. It is shown that the C6 was released by diffusing from the film and dissociation of the BCM layers. And two different release mechanism simultaneously affected insoluble drug release profile^11^,^49^. As a result, in the pH 7.4 PBS buffer (Fig. 7a), the drug release of the films lasted for 6, 52, and 108 h, whereas all the films exhibited relatively short release lasting up to 6 and 38 h in the pH 2 PBS buffer (Fig. 7b). This is a general method for the controlled release of drugs from lbl assembled films, which are used for targeted drug delivery to specific internal environments.

Based on the previous publications, the GO sheet in the lbl assembled multilayer films interrupts drug transfer^28^. In addition, multilayer films containing GO layers were used in the sustained drug release films. On the other hand, when the surface of film has high roughness, the film has large surface area. So, it is easily occurs that the permeation of a liquid solvent into the inner layer of rough or porous multilayer film. Then, the solvent-soluble drug in the film rapidly diffuses out. For these reason, it was expected that the bPEI/BCM film which had higher roughness than GO/BCM film had rapid drug release. However, owing to the lower proportion of the GO layer than the proportion of the BCM layer in the overall film, as mentioned above, the barrier effect imparted by the GO sheet was not effective in our film. Besides, when the films degradation occurred by decreasing interaction between interfaces of each layer, the BCM on GO sheet dissociated from film at once, whereas the BCM on bPEI layer partially disassembled (Fig. 7c). Therefore, the release rate of the drug from the (GO/BCM)_7_ film was more rapid than the release of the drug from the (bPEI/BCM)_7_ film under both pH 7 and pH 2 conditions. Interestingly, we expected the bPEI/BCM film with inserted GO layers (e.g., bPEI/BCM/GO/BCM) to have a release rate midway between those of the bPEI/BCM and GO/BCM films. However, the (bPEI/BCM/GO/BCM)_3.5_ film exhibited the highest release rate under both pH conditions.

## Conclusion

In summary, we have demonstrated the effect of pH on the properties of multilayer thin films consisting of bPEI, GO, and BCM assembled via the lbl method for drug delivery system applications. The multilayer thin films were fabricated as a result of electrostatic interactions between amphiphilic BCM of PS*-b-*PAA and bPEI or GO. We investigated the effect of pH on the film properties including thickness, roughness, topology and adsorption using a profilometer, AFM, SEM and QCM. We also related different film properties to the effect of pH variation on structural change. Especially, we obtained results conflicted with previous research of sustained release by GO capping layer. Rather, inserting GO layer into multilayer film for drug delivery induced burst drug release. We anticipate that these results will be used to control the fabrication of drug delivery films consisting of various materials under various pH conditions and to optimize the release rate of drugs from drug delivery films by modification of the film structure. For instance, we can obtain the uniformed and strengthened film consisting of micelle and polymer by using graphene oxide layer. Also, it can be possible that the stimuli-responsive film which have more rapid drug release by using drug incorporated micelle and an electric stimuli-responsive graphene oxide sheet. Finally, this approach will allow the control of the properties of lbl assembled coatings in various biomedical applications since without limitation of substrate for lbl assembly.

## Additional Information

**How to cite this article**: Han, U. *et al*. Effect of pH on the structure and drug release profiles of layer-by-layer assembled films containing polyelectrolyte, micelles, and graphene oxide. *Sci. Rep.* **6**, 24158; doi: 10.1038/srep24158 (2016).

## Supplementary Material

Supplementary Information

## Figures and Tables

**Figure 1:**
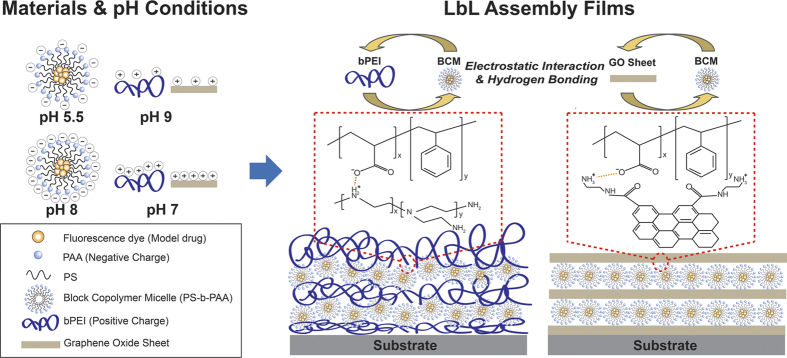
Schematic illustration of the materials used, pH-dependent charge density of the layer by layer (lbl) assembly building block (left), and bPEI/BCM and GO/BCM multilayer films assembled by the lbl assembly method with electrostatic interactions and hydrogen bonding (right).
