# Supplementary material for: Effect of pH on the structure and drug release profiles of layer-by-layer assembled films containing polyelectrolyte, micelles, and graphene oxide
Source: Sci Rep. 2016 Apr 7;6:24158. doi: 10.1038/srep24158 (PMC4823712; doi:10.1038/srep24158)
Supplement: Supplementary Information [file srep24158-s1.doc]

**Supplementary information**

Effect of pH on the structure and drug release profiles of layer-by-layer assembled films containing block copolymer micelles, and graphene oxide

Uiyoung Han^1^, Younghye Seo^1^, and Jinkee Hong^1,*^

^1^ School of Chemical Engineering & Materials Science, College of Engineering, Chung-Ang University, 47 Heukseok-ro, Dongjak-gu, Seoul 156-756, Republic of Korea

**Supplementary Information Figure S1**


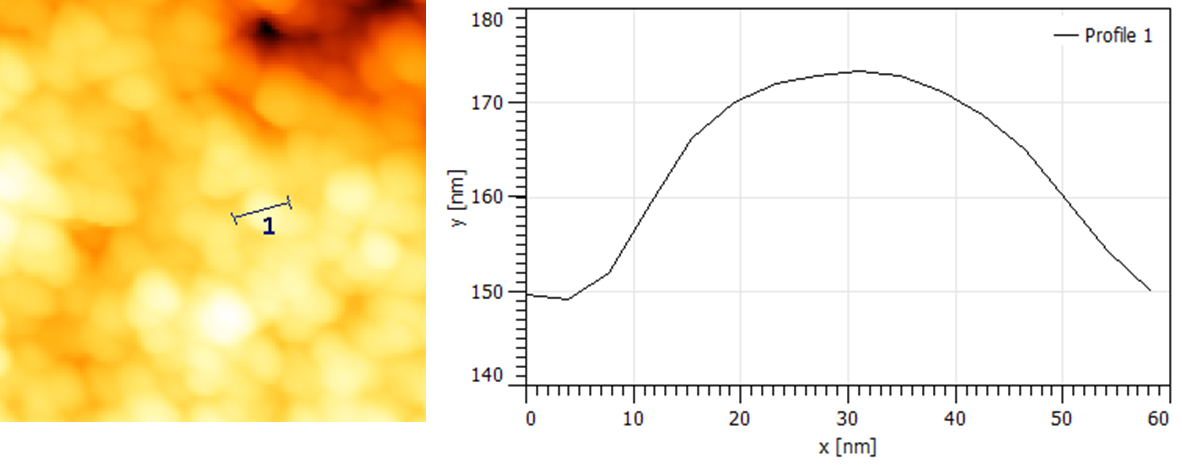


**Figure S1.** AFM image of (bPEI/BCM) multilayer film shows the size of BCM (approximately, 52 nm) adsorbed on film.

**Supplementary Information Figure S2**


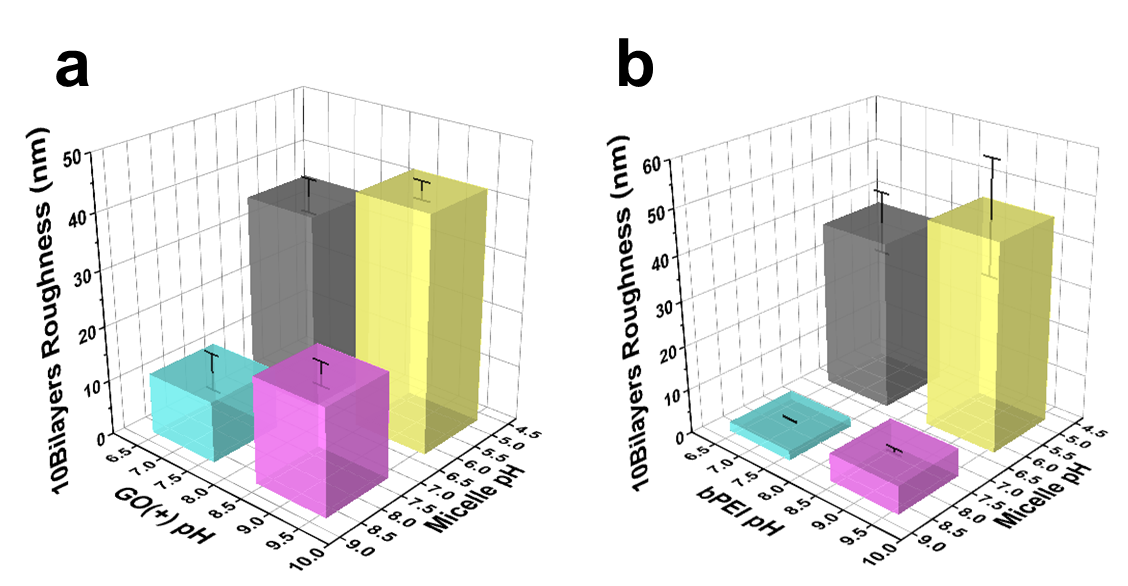


**Figure 2S.** Each matrix shows the RMS surface roughness of (a) (bPEI/BCM)_10_ film and (b) (GO/BCM)_10_ film fabricated in bPEI, GO, and BCM solution at different pH conditions. The RMS values were measured by profilometry.

**Supplementary Information Figure S3**


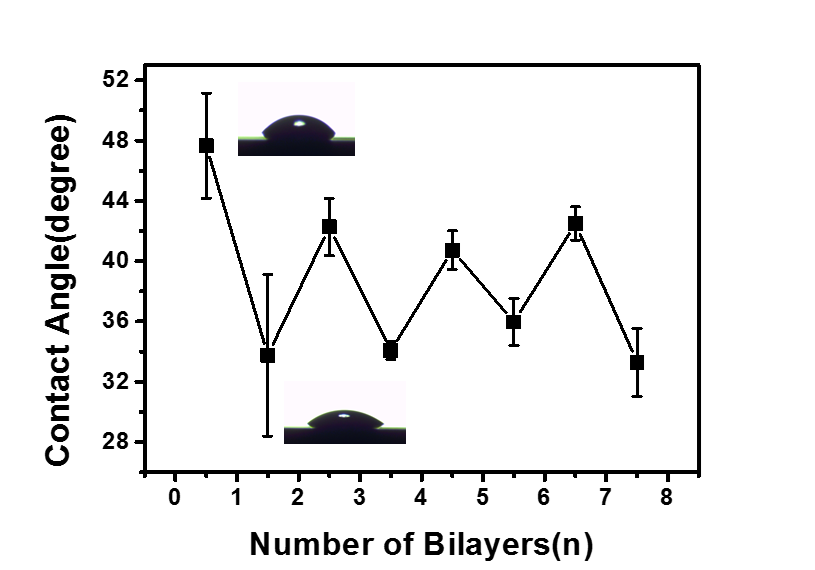


**Figure 3S.** The graph shows variation of contact angle in fabricating (bPEI/BCM/GO/BCM)_n/2_ film. When the top layer is bPEI (n=0.5, 2.5, 4.5 and 6.5) the contact angle of film surface is higher value than when the top layer is GO (n=1.5, 3.5, 5.5 and 7.5). It shows consecutive lbl assembly of (bPEI/BCM/GO/BCM) multilayer film.

**Supplementary Information Figure S4**


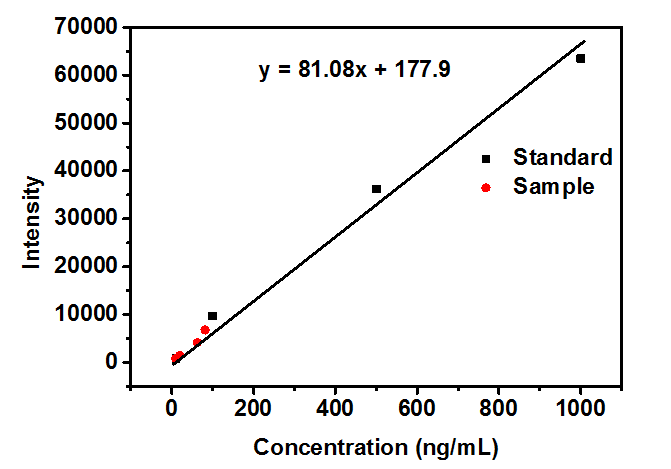


**Figure S4.** Concentration of each release sample and standard curve established by measuring the photoluminescence. The standard curve of x-axis was the C6 concentration (1000, 500, 100 and 10 ng/mL) and the y-axis was values of PL intensity.

**Supplementary Information Figure S5**


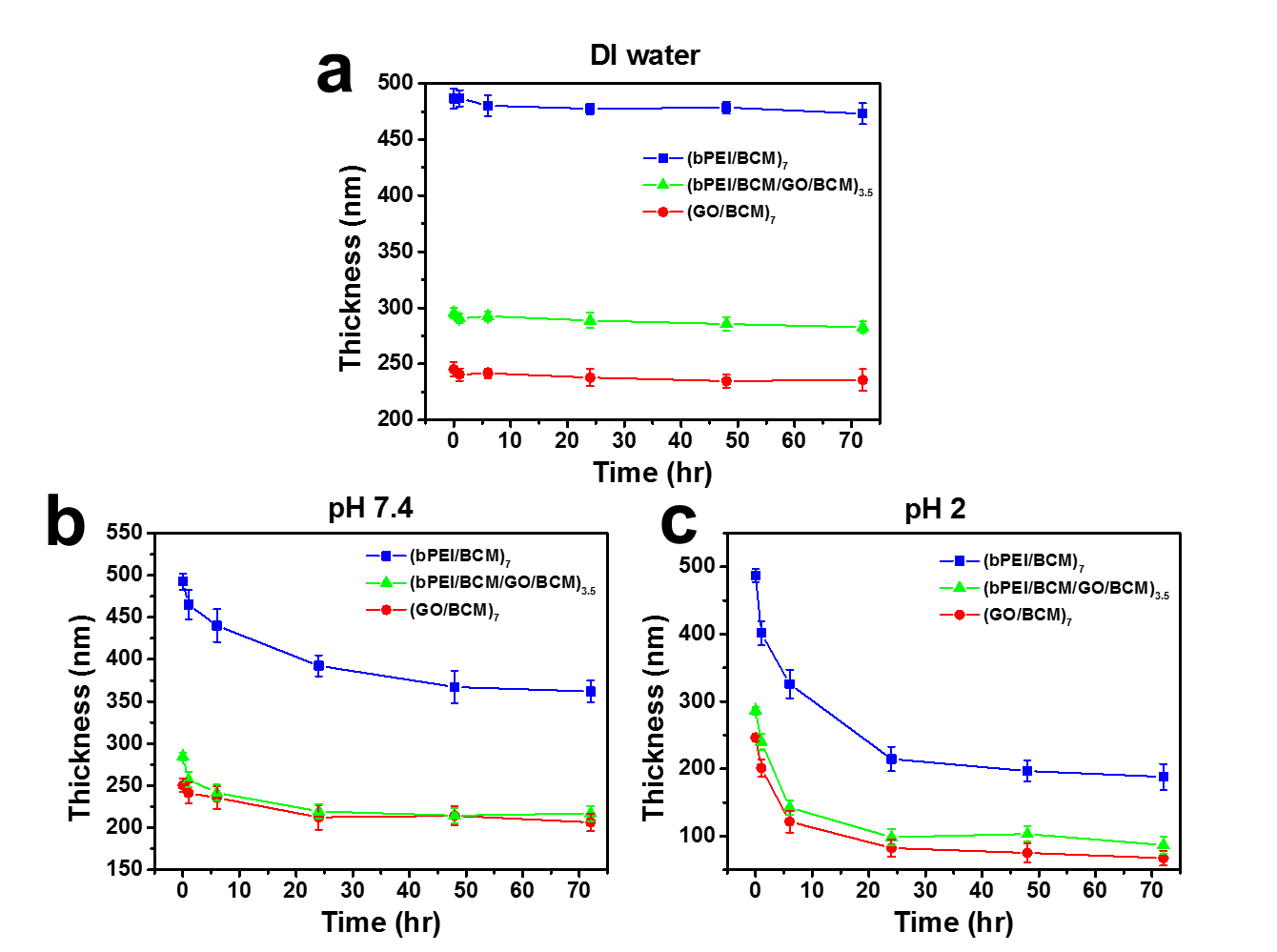


**Figure S5.** The stabilities of (bPEI/BCM)_7_, (bPEI/BCM/GO/BCM)_3.5_ and (GO/BCM)_7_ films in (a) distilled water and (b, c) pH 7.4 & 2 PBS/EtOH(2:1).
